# First-Principles Study of Sodium Intercalation in Crystalline Na_*x*_ Si_24_ (0 ≤ *x* ≤ 4) as Anode Material for Na-ion Batteries

**DOI:** 10.1038/s41598-017-05629-x

**Published:** 2017-07-13

**Authors:** Unai Arrieta, Nebil A. Katcho, Oier Arcelus, Javier Carrasco

**Affiliations:** CIC Energigune, Parque Tecnológico de Álava, C/Albert Einstein 48, 01510 Miñano, Vitoria, Álava Spain

## Abstract

The search for Si-based anodes capable of undergoing low volume changes during electrochemical operation in rechargeable batteries is ample and active. Here we focus on crystalline Si_24_, a recently discovered open-cage allotrope of silicon, to thoroughly investigate its electrochemical performance using density functional theory calculations. In particular, we examine the phase stability of Na_*x*_Si_24_ along the whole composition range (0 ≤ *x* ≤ 4), volume and voltage changes during the (de)sodiation process, and sodium ion mobility. We show that Na_*x*_Si_24_ forms a solid solution with minimal volume changes. Yet sodium diffusion is predicted to be insufficiently fast for facile kinetics of Na-ion intake. Considering these advantages and limitations, we discuss the potential usefulness of Si_24_ as anode material for Na-ion batteries.

## Introduction

Li-ion rechargeable batteries are the energy storage system for current commercial portable electronics. However, the demand is swiftly moving to future long-term and large-scale applications (*e*.*g*., electric vehicles and stationary storage for electric grids). This raises serious sustainability concerns associated to the massive use of Li-based energy storage technologies, such as limited lithium resources and their steeply increasing price^1, 2^. In this context, the high abundance and low cost of sodium make Na-based batteries an attractive alternative to Li-ion batteries^3^. This, along with the environment-friendly nature of sodium production, has triggered a flurry of interest, and research on new Na-based electroactive materials has gained momentum in the past few years^2, 4–12^. Yet limited progress has been achieved from the viewpoint of identifying suitable anode materials for Na-ion batteries.

For decades, the long road to Li-ion power has gathered extensive knowledge on the redox chemistry between lithium and a wide range of hosts materials (see, *e*.*g*., ref. 13 for a brief review). Given the chemical similarity between lithium and sodium, these accomplishments might be seen as a highway towards the design of equivalent Na-based materials. However, the more we progress in the quest for better Na-based materials, the more we become aware that this approach must be taken with caution, especially in the case of anodes. For instance, graphite is the most common anode-active compound used in today’s Li-ion batteries^14, 15^. But graphite is electrochemically much less active towards sodium insertion^16, 17^. Nowadays one of the most intensively studied anode materials for Li-ion batteries is silicon, which offers one of the highest theoretically possible specific capacities (*e*.*g*., Li_22_ Si_5_ with 4199 mAh/g, more than ten times larger than that of graphite)^18^. A number of studies have demonstrated experimentally the electrochemical lithiation of silicon (see, *e*.*g*., refs 19 and 20), but again all efforts to electrochemically insert Na ions into stand-alone silicon have failed so far^21–23^.

Si-based anodes are certainly very desirable materials for Na-ion batteries^24–26^. Silicon is the second-most abundant element in the Earth’s crust and it is environmentally benign. This makes silicon the ideal match for sodium in order to attain sustainable and inexpensive batteries. The main problem associated to the electrochemical ion insertion of silicon is that (de)intercalation is accompanied with large volume changes and mechanical stresses that weaken the host lattice. This degrades the morphology of the anode during cycling, with large irreversible capacity losses^27–29^. The problem is specially dramatic for sodiation, which results in a larger volume expansion than lithiation due to the larger size of sodium. For instance, recent *ab initio* molecular dynamics simulations showed that the volume expansion of amorphous Li-Si alloys increases linearly with lithium content up to 160%, whereas Na-Si alloys expand to about 230% for the same alkali content^30^.

Forming a solid-state solution of crystalline silicon under electrochemical conditions is one of the best strategies to preserve the morphology of the anode during cycling. Indeed, lithium has been inserted electrochemically into crystalline silicon, with Li_3.75_Si as the most Li-rich phase known to date^31^. Crystalline Na-Si alloys have also been reported (*e*.*g*., NaSi^32^), however, as previously noted, crystalline silicon is inactive towards electrochemical sodium insertion^21–23^. Finding novel crystalline silicon phases beyond cubic-diamond (silicon’s most common form) that are capable of intercalating sodium is, therefore, of the outmost technological relevance. Fortunately, silicon shows a rich variety of bulk phases and allotropes^33–36^. And a thoroughly exploration of such complex free-energy landscape might yield very fruitful results. Open-cage structures are particularly compelling, since, in principle, they could easily accommodate alkali ions inside the cages. Actually, it has been found that several classes of silicon clathrates evidence negligible volume changes during electrochemical lithium insertion^37^. Yet little is known about the corresponding sodium counterparts.

Interestingly, the synthetic strategies that led to the discovery of many silicon clathrates and other open-cage structures rely on Na-based precursors (*e*.*g*., Na_4_Si_24_^38^, Na_*x*_Si_136_^39^, and Na_8_Si_46_^39^), which suggests that such compounds could show high sodium intercalation capacities and fast ion mobility^40^. This, together with the fact that often these compounds are metallic^41, 42^, makes open framework structures of silicon attractive candidates for anode materials. Motivated by this, Marzouk *et al*.^43^ have recently explored the insertion of sodium into different allotropic forms of crystalline Na_*x*_Si_24_ (with *x* = 1, 2, 3, 4, 5, and 6): (i) the experimentally observed open-cage Si_24_^33–38^ and (ii) additional metastable allotropes found computationally using evolutionary metadynamics. They showed that sodiation in all these systems is energetically favourable up to four sodium atoms per Si_24_, with small volume expansions and low intercalation potentials. These results provide basic evidence for the potential benefits of using Si_24_ as anode material. Follow-up studies to fully understand the electrochemical behaviour of such compounds are therefore timely and highly desirable.

Here, we focus on the most stable allotrope Si_24_ and thoroughly assess its potential electrochemical performance as anode material for Na-ion batteries. To this end, we carried out a series of density functional theory (DFT) calculations to examine a range of key properties during battery operation. First, we computed the equilibrium phase diagram of Na_*x*_Si_24_ (0 ≤ *x* ≤ 4) at 0 K in order to identify possible phase separation and solid solution regions. Second, we analysed volume and voltage changes along the whole composition range. Then, sodium mobility within the open-cage network of Si_24_ was explored by computing Na-ion diffusion barriers. Finally, in light of our results, we discuss the advantages and drawbacks of Si_24_-based insertion anode for Na-ion cells and how future research directions might help to improve this class of anode materials.

## Methods and computational details

We performed spin-polarized DFT calculations as implemented in the code VASP (version 5.4.1)^44, 45^. Projector augmented wave potentials^46^ were employed to treat valence-core interactions, whereas Na (2p, 3 s) and Si (2 s, 2p) valence electrons were expanded in plane-waves with a cut-off energy of 600 eV. The Perdew–Burke–Ernzerhof (PBE)^47^ exchange–correlation functional was used together with a Monkhorst–Pack grid centred at the Γ point with at least 8 × 4 × 4 *k*-point sampling per 1 × 1 × 1 unit cell. The structures were fully optimized (cell parameters, volume cells, and atomic positions) with a residual force threshold of 0.02 eV/Å^−1^. These computational settings guaranteed a tight convergence in total energies (ca. 3 meV per formula unit) and lattice parameters (differences are less than 2%). Additionally, we confirmed that the cut-off energy of 600 eV was sufficiently large to mitigate the problems of Pulay stress and incomplete basis set associated to changes of the volume in plane-waves calculations.

**Figure 1:**
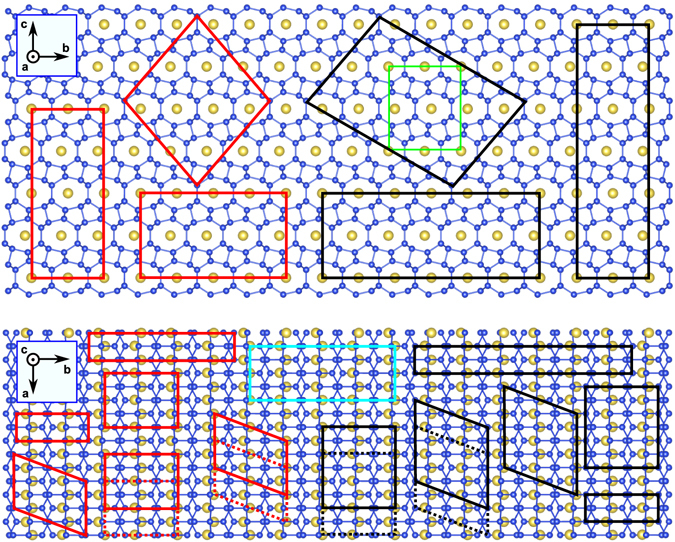
Structure of Na_4_Si_24_ projected in *b*-*c* and *a*-*b* planes. The parallelograms indicate all of the supercells used to generate different Na_*x*_Si_24_ configurations when sodium atoms are removed from the structure. Red, black, and light blue parallelograms correspond to supercells containing 8, 12, and 16 formula units, respectively. The green rectangle shows the unit cell, with 4 formula units. Blue and yellow balls stand for silicon and sodium atoms, respectively.

Taking as a reference the pristine Si_24_ unit cell with three crystallographic non-equivalent Si positions (space group *Cmcm*)^38^, we simulated a range of Na_*x*_Si_24_ (0 ≤ *x* ≤ 4) systems using supercells containing up to 16 formula units (Na_*x*/4_Si_6_) as shown in Fig. 1. We used the cluster assisted statistical mechanics (CASM) code^48^ to generate all of the possible sodium and sodium vacancy ($\square$) arrangements within each supercell. By so doing, we thoroughly took into account the configurational order/disorder associated with different Na-$\square$ arrangements for a given sodium concentration. However, given the large number of possible Na-$\square$ configurations for most of the Na concentrations, we selected only a fraction of them to be actually computed using DFT. The selection was driven by a fitting procedure using a cluster expansion scheme as implemented in CASM^48^. Overall, we computed with DFT a total of 235 different configurations and the specifically investigated Na_*x*_Si_24_ compositions were x = 0.000, 0.250, 0.333, 0.500, 0.667, 1.000, 1.333, 1.500, 1.667, 2.000, 2.333, 2.500, 2.667, 3.000, 3.333, 3.500, 3.667, and 4.000. The DFT runs of the entire set of configurations (all input and output files) are hosted by NoMaD (Novel Materials Discovery) Repository in the following link http://dx.doi.org/10.17172/NOMAD/2017.03.17-2.

The transition-state structures and activation barriers for Na-ion diffusion were calculated using the climbing image nudged elastic band (CI-NEB) method^49^ with seven images along the migration pathway. The calculations were performed at constant volume using the optimized lattice parameters of the lowest-energy Na_0.333_Si_24_ and Na_3.667_Si_24_ configurations. The inclusion of van der Waals (vdW) forces into plain DFT can play a role in the magnitude of the computed activation energies for ion diffusion in sparse matter (see, *e*.*g*., refs 50 and 51). Therefore, we used the vdW-inclusive DFT-D3 method with a Becke-Johnson damping function (DFT-D3BJ)^52^, which has shown good accuracy to describe vdW layered electroactive materials as compared with other vdW-inclusive approaches^53^, to test the effect of vdW forces on our computed activation energies. We found that the impact of vdW forces is negligible in Si_24_, with energy differences less than 2 meV between PBE and D3BJ.

## Results

### Phase stability as a function of sodium content

We examined the relative stability of Na_*x*_Si_24_ with sodium concentrations ranging from *x* *=* 0 to *x* *=* 4. First, we computed the total energy of different Na-$\square$ arrangements with composition *x*; Table S1 (Supplementary Material) summarises the ground state and second most stable structures for each *x*, including their optimized cell parameters. Then, for each configuration, the corresponding formation energy per formula unit was calculated as

1 $${{\rm{\Delta }}}_{{\rm{f}}}E={E}_{{{\rm{Na}}}_{x}{{\rm{Si}}}_{24}}-\frac{x}{4}{E}_{{{\rm{Na}}}_{4}{{\rm{Si}}}_{24}}-(1-\frac{x}{4}){E}_{{{\rm{Si}}}_{24}},$$

where ${E}_{{\rm{N}}{a}_{x}S{i}_{24}}$ is the total energy of a given Na_*x*_Si_24_ configuration per Na_*x*/4_Si_6_ formula unit, ${E}_{{\rm{N}}{a}_{4}S{i}_{24}}$ is the total energy of the fully sodiated phase per NaSi_6_ formula unit, and ${E}_{{\rm{S}}{i}_{24}}$ is the total energy of pristine Si_24_ per Si_6_ formula unit. Negative formation energies correspond to stable Na_*x*_Si_24_ structures.

**Figure 2:**
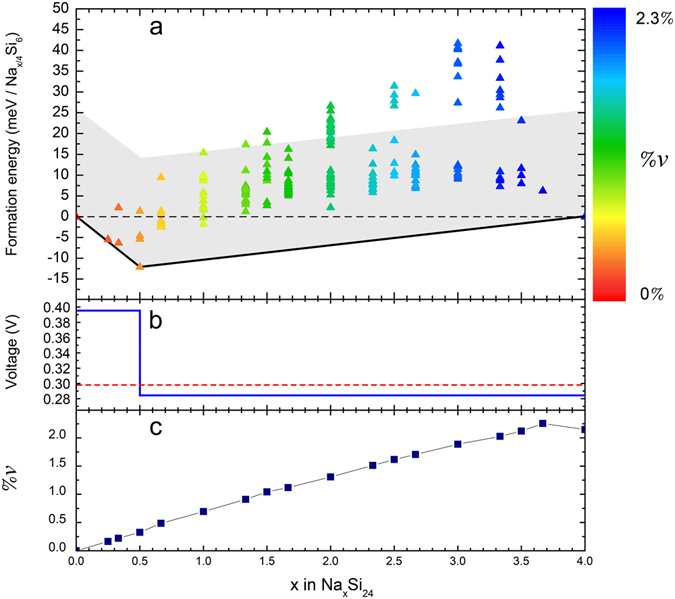
(**a**) Calculated formation energies as a function of sodium content in Na_*x*_Si_24_ structures with different Na-$\square$ arrangements. The grey shaded area indicates formation energies within 25.6 meV (*kT* at 25 °C) above the hull line. (**b**) Computed zero-temperature voltage curve (solid blue line) and average cell voltage (dashed red line). (**c**) Calculated volume change (%*v*) for each Na_*x*_Si_24_ ground state structure as a function of sodium content. The colour gradient scale in (**a**) shows the volume change for all considered structures.

Figure 2(a) shows Δ_f_E as a function of sodium content and includes the corresponding convex hull. The convex hull envelopes all the points in the plot by connecting the lowest-energy structures with straight lines. Given two inflexion points in the convex hull (stable structures) at sodium concentrations *x*_*i*_ and *x*_*j*_, the straight line connecting them indicates a tendency of any intermediate Na_*x*_Si_24_ composition (*x*_*i*_ < x < *x*_*j*_) for phase separation into a fraction (*x*_*j *_− *x*)/(*x*_*j *_− *x*_*i*_) of ${{\rm{Na}}}_{{x}_{i}}{{\rm{Si}}}_{24}$ and a fraction (*x* − *x*_*i*_)/(*x*_*j*_ − *x*_*i*_) of ${{\rm{Na}}}_{{x}_{j}}{{\rm{Si}}}_{24}$, according to the application of the lever rule. The convex hull can therefore be interpreted as the zero-temperature mixing free energy of the system (*i*.*e*. neglecting configurational entropy).

We found the formation of a stable intermediate phase at *x* = 0.5 that governs the convex hull (Figure S1 in Supplementary Material). Accordingly, sodiation of Si_24_ is predicted to occur through two successive phase transitions: (i) between Si_24_ and Na_0.5_Si_24_, and (ii) between Na_0.5_Si_24_ and Na_4_Si_24_. Similarly, desodiation of Na_4_Si_24_ should follow the inverse process. However, notice that the formation energies are very low along the whole composition range, and they are easily accessible at room temperature (*kT* = 25.6 meV at 25 °C), especially below *x* = 2. In total 189 formation energies are below *kT* with respect to the hull line (grey shaded area in Fig. 2(a)). This provides accessible low-energy Na-$\square$ configurations through intermediate Na_*x*_Si_24_ structures and, therefore, it suggests the formation of a solid solution regime along the whole range of sodium compositions. These results are in agreement with experimental observations^38^ showing that the thermal release of sodium from Na_4_Si_24_ occurs spontaneously at temperatures as low as 47 °C, with complete sodium removal after eight days under vacuum at 127 °C.

### Average voltage versus Na/Na^+^

DFT calculations can also be used to predict average equilibrium voltages (sodiation potential) as a function of capacity, *V*(*x*)^54^. The average voltage for sodium compositions between *x*_*i*_ and *x*_*j*_ (with *x*_*i*_ < *x*_*j*_) is given by the negative change in the corresponding Gibbs free energy (Δ*G* ^*ij*^) per charge carrier. For zero-temperature calculations, Δ*G* ^*ij*^ (≡Δ*E* ^*ij*^ + *P*Δ*V* ^*ij*^ + *T*Δ*S* ^*ij*^) can be approximated by simply considering the change in total energy (Δ*E* ^*ij*^)^54^, neglecting zero-point energy contributions and the term *P*Δ*V* ^*ij*^:

2 $$V(x)=-\frac{\Delta {G}^{ij}}{({x}_{j}-{x}_{i})F}\approx -\frac{[{E}_{{{\rm{Na}}}_{{x}_{j}}{{\rm{Si}}}_{24}}-{E}_{{{\rm{Na}}}_{{x}_{i}}{{\rm{Si}}}_{24}}-({x}_{j}-{x}_{i}){E}_{{\rm{Na}}(s)}]}{({x}_{j}-{x}_{i})e},$$

where *F* is Faraday’s constant, *e* is the electron charge, ${E}_{{{\rm{Na}}}_{{x}_{i}}{{\rm{Si}}}_{24}}$ $({E}_{{{\rm{Na}}}_{{x}_{j}}{{\rm{Si}}}_{24}})$ is the total energy of ${{\rm{Na}}}_{{x}_{i}}{{\rm{Si}}}_{24}$ $({{\rm{Na}}}_{{x}_{j}}{{\rm{Si}}}_{24})$ ground state structure, and *E*_Na(s)_ is the total energy (per atom) of bulk *bcc* sodium. The approximation is indeed good at room temperature as well, since finite-temperature entropic effects are only expected to smoothen the voltage curve.

Figure 2(b) shows the computed voltage curve along the minimum energy path of formation energies (Fig. 2(a)). Our computed average voltage between the fully sodiated (*x* = 4) and desodiated (*x* = 0) phases is 0.30 V. In addition, we predict a small voltage jump of 0.11 V at *x* = 0.5, which corresponds to the formation of the intermediate Na_0.5_Si_24_ structure. These results are consistent with the intercalation potentials reported by Marzouk *et al*.^43^ for Na_1_Si_24_, Na_2_Si_24_, Na_3_Si_24_, and Na_4_Si_24_. In particular, their average voltage considering these four structures is also ~0.30 V.

### Volume expansion as a function of sodium content

In Fig. 2(a) we used a colour gradient scale to follow the volume change (%*v*) associated to each Na_*x*_Si_24_ structure. Taking as a reference the desodiated pristine Si_24_ phase, the volume change was calculated as

3 $$ \% v=100\cdot \frac{{v}_{x}-{v}_{0}}{{v}_{0}},$$

where *v*_*x*_ and *v*_0_ are the volumes of the sodiated Na_*x*_Si_24_ and pristine Si_24_ phases, respectively.

Our results indicate that the (de)sodiation process along the whole composition range is accompanied by small volume changes (less than 2.3%). Further analysis shows that, considering only the lowest-energy Na_*x*_Si_24_ structure for each *x* value, there is a systematic volume expansion when increasing the sodium content, as shown in Fig. 2(c). This is in good agreement with previous DFT calculations and experimental evidence for a limited number of sodium concentrations^38^. Interestingly, the volume changes associated to different Na-$\square$ arrangements within a given sodium content are generally very small (less than 0.2%). This suggests that the geometrical flexibility of the silicon framework is independent of how sodium and sodium vacancies get distributed. This property is, in principle, beneficial to avoid possible mechanical stresses which could be associated to the eventual visit of high-energy Na-$\square$ configurations during electrochemical operation.

The observed small volume changes are a direct consequence of the low-density and cage-like structure of allotrope Si_24_, which facilitates that Na ions enter and leave the cages with minimal distortion of the open-framework arrangement of Si. Essentially, Si_24_ presents cages large enough to easily accommodate guest Na ions.

### Sodium diffusion kinetics

We examine now the migration of sodium in Na_*x*_Si_24_ at dilute (*x* = 0.333) and concentrated (*x* = 3.667) regimes. These two concentration limits allow us to easily assess the effect of Na-Na interactions on diffusion barriers. In the dilute limit, the diffusing sodium ion is completely surrounded by sodium vacancies and the impact of Na-Na interactions on the diffusion barrier is minimal. In contrast, by imposing a high sodium concentration Na-Na interactions are maximized. In principle, diffusion barriers along intermediate sodium concentrations are expected to range between these two limits.

**Figure 3:**
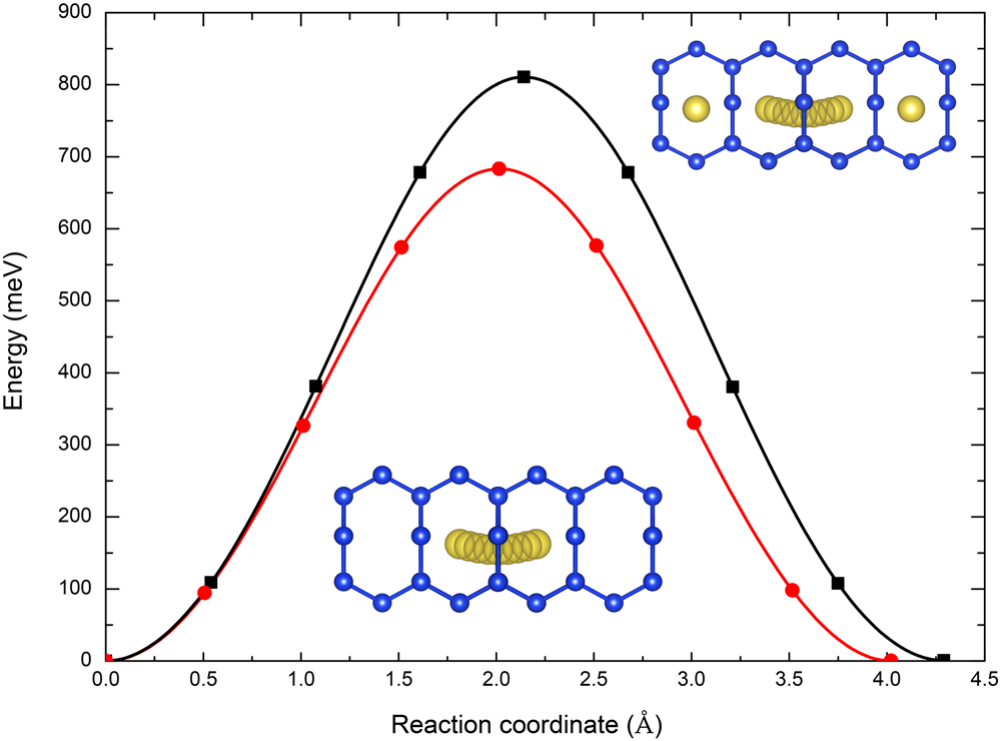
Migration energy profiles for sodium diffusion in Na_0.333_Si_24_ (red circles) and Na_3.667_Si_24_ (black squares). The lines connecting the points correspond to a spline fitted to the calculated CI-NEB energies. The insets show the atomic structures of the diffusing sodium ion along the minimum energy paths for Na_0.333_Si_24_ (bottom) and Na_3.667_Si_24_ (top).

Sodium migration in the open framework of Si_24_ corresponds to a hop path between two low-energy sites along the *a* lattice axis, within the linear channels formed by eight-member silicon rings (see insets in Fig. 3). Sodium diffusion along the alternative *b* and *c* lattice axes is blocked by the presence of too narrow six- and five-member silicon rings, respectively. The computed migration energy profiles at *x* = 0.333 and *x* = 3.667 using CI-NEB calculations are shown in Fig. 3. The diffusion barrier for the dilute limit is 683 meV, and it increases to 811 meV in the case of high concentration (Table 1). Further analysis shows that, for the transition state structure at *x* = 0.333, the eight-member ring along the diffusion pathway expands up to 0.46 Å with respect to the initial positions of the silicon atoms. For *x* = 3.667, however, the presence of sodium atoms in nearby cages hinders the outward relaxation of silicon atoms, with an expansion of the eight-member ring at the transition state structure of ≤0.43 Å. This geometrical constrain in turn explains the observed trend in the diffusion barriers when moving from the dilute to concentrated regime.

**Table 1:** Computed diffusion barriers for Na migration using CI-NEB calculations.

x in Na_*x*_Si_24_	Diffusion barrier (meV)
0.333	683
0.667	811

## Discussion

Our first-principles computations show that crystalline Na_*x*_Si_24_ exhibits useful electrochemical properties and is, therefore, an interesting anode material for Na-ion batteries. First, Na_*x*_Si_24_ forms a solid solution along the whole range of sodium compositions. Secondly, it shows negligible volume expansion upon sodium insertion. Thirdly, the operating voltage versus Na/Na^+^ is conveniently low.

The formation of a solid solution implies that Na_*x*_Si_24_ will evolve through a single-phase regime, rather than by nucleation and growth of other phases, which has significant consequences for the (de)sodiation process. Essentially, Na_*x*_Si_24_ grains should insert or remove sodium as rapidly as their bulk and interfacial diffusion kinetics allow. Therefore, during electrochemical operation the potential required to drive (de)sodiation will be nearly constant and low. In addition, the potential negative effects of phase separation (*e*.*g*., mechanical stresses, sluggish kinetics, or problems of irreversibility) are effectively avoided. Notice, however, that the equilibrium state of the Na_*x*_Si_24_ system is actually governed by a ground state at *x* = 0.5 (Fig. 2(a)). Hence, at sufficiently large relaxation times (thermodynamic conditions), a Na_*x*_Si_24_ anode should evolve to its equilibrium two-phase state of Si_24_/Na_0.5_Si_24_ or Na_0.5_Si_24_/Na_4_Si_24_ depending on the initial sodium content.

The main drawback of current Si-based anode materials is their massive volume change during ion insertion and extraction. Hence, even though the formation of a solid solution is beneficial, it should be accompanied by minimal volume changes along the whole sodium composition range. Our results indicate that the structural retaining ability of Na_*x*_Si_24_ is indeed excellent, with a maximum volume expansion of 2.3% along the whole sodium composition range. This is in contrast to the massive volume expansion of other binary sodium compounds, such as *a*-Na_0.76_Si (%*v* = 114%)^55^, *a*-NaSi (%*v* = 230%)^30^, *a*-Na_3_Sb (%*v* = 293−390%)^56, 57^, or *a*-Na_3.75_Sn (%*v* = 480%)^30^. Interestingly, the volume changes of Na_*x*_Si_24_ are even smaller than those reported for some low-strain sodium titanate systems (*e*.*g*., %*v* = 2.8% for Na_2_Ti_7_O_15_-Na_4_Ti_7_O_15_^58^ and %*v* ≈ 5% for Na_2_Ti_3_O_7_-Na_4_Ti_3_O_7_^59^). Na_*x*_Si_24_ is therefore expected to offer a very competitive structural function as it cycles sodium in and out.

The average voltage of a suitable anode material should be low (typically around 0–1 V versus Na/Na^+^) to maximize the overall cell potential and in turn its energy density. For instance, promising sodium anodes such as NiP_3_^60^, disodium terephthalate (Na_2_C_8_H_4_O_4_)^61, 62^, and some oligomeric-Schiff bases^63, 64^ present operating voltages of 0.3–0.8 V, 0.4–0.6 V, and 0.6–1.2 V versus Na/Na^+^, respectively. Electrodes with voltages too close to 0 V are indeed highly prone to sodium plating on charge. This issue makes them unsafe and, in general, operating voltages slightly above 0 V are desirable. The predicted average voltage of Na_*x*_Si_24_ (0.30 V) and its flat profile are therefore beneficial to improve safety as anode material while offering a good energy density.

All of these predicted properties are certainly encouraging, specially taking into account that the search of open framework allotropes of silicon as electrode materials for batteries is at its early stage. Yet, from a critical viewpoint, further developments are required in order to advance such compounds towards actual practical applications. For instance, our calculations pointed out a need for enhancing sodium mobility in bulk Na_*x*_Si_24_. The computed diffusion barriers (683–811 meV) are lower than that of bulk silicon (~1000 meV)^65^, but about twice larger than the values reported for *a*-Na_0.76_Si (380 meV)^55^ or *a*-NaSi (310 meV)^30^. In general, good ionic conductors should show bulk diffusion barriers lower than 400 meV^66^.

Another issue is the moderate capacity of Si_24_. Considering the volume of desodiated Si_24_ as a reference, the theoretical volumetric capacity is 337 mAh/cm^3^, which is far from the values of bulk sodium (~1100 mAh/cm^3^) or NaSi (~950 mAh/cm^3^), but similar to that of, for example, hard carbon (Na_0.81_C_6_ with ~375 mAh/cm^3^)^67^. The theoretical gravimetric capacity of 159 mAh/g (assuming 4 sodium ions are reversibly inserted) is similar to that delivered by some sodium titanates and titanium phosphates that have also been proposed as anodes for Na-ion batteries^7^. Still, these values are quite behind the ~300 mAh/g of reversible capacity offered by hard carbon, which is currently a very attractive candidate material due to its low cost. Inserting additional sodium ions into Si_24_ structures would be a good strategy to mitigate this issue. Indeed, Marzouk *et al*.^43^ computationally explored such possibility by considering alternative stable forms of Si_24_. By breaking different Si-Si bonds, they were able to generate additional open cages within each of those allotropes and in turn rearrange the unit cell space to accommodate more alkali ions. This virtual experiment showed that those new allotropes could indeed allocate up to 7 lithium ions instead of only 4, leading therefore to a substantial capacity increase. In contrast, the results were less successful for the sodium counterparts due to the larger size of sodium as compared to lithium; insertion beyond 4 sodium ions was shown to be unfavourable. However, given the rich free-energy landscape of silicon, these studies open an interesting molecular-level strategy for the future design of improved crystalline allotropes.

Finally, it is important to notice that synthesizing low-cost Si_24_ is of paramount relevance for their actual success as anode materials. Although Si_24_ is stable at ambient conditions, its synthesis involves high pressures^38^. Likewise, current production of other open-framework silicon compounds is also expensive. Therefore, the development of new cost effective synthesis methods are mandatory. Fortunately, silicon is the focus of a number of large semiconductor industries, which makes such research attractive to them. Another reason to be optimistic is that other Si-based anode materials, firstly synthesized at high temperatures and high pressures, have then been prepared by more simple and affordable techniques. In particular, Duveau *et al*.^68^ reported that SiP_2_ (first prepared at >1000 °C and 1–4 GPa) can be obtained by ball milling of silicon and phosphorous powders at room temperature.

## Conclusions

In this work we focused on the recently discovered open-cage allotrope Si_24_ and, using DFT calculations, we assessed several of its thermodynamic and kinetic properties of relevance for the potential use as anode material for emerging Na-ion technologies. Our results shed light into the phase stability of Na_*x*_Si_24_ along the whole composition range (0 ≤ *x* ≤ 4), predicting the formation of a beneficial solid solution at room temperature. In addition, we found that the (de)sodiation process is accompanied by low volume changes (less than 2.3%) and low average voltage (0.30 V). These characteristics make Si_24_ an interesting anode material for Na-ion batteries.

However, we also identified a series of current limitations and concerns for its practical use. First, the computed sodium diffusion barriers in bulk Na_*x*_Si_24_ indicate that sodium ions are expected to be only moderately mobile, likely leading to too sluggish kinetics for competitive electrochemical performance. Secondly, the specific capacity of Si_24_ is acceptable, but clearly lower than that of other competing materials such as hard carbon. And finally, current Si_24_ synthesis techniques need to improve in terms of cost effectiveness.

Overall, while Si_24_ shows interesting opportunities as anode for Na-ion batteries, further improvements are required to unveil the full potential of such class of materials. Aware of the predicted advantages and current limitations of Si_24_, we put forward that the superior volume change behaviour of Si_24_ makes this material suitable for applications where mechanical reliability and duration are more important than cost or high capacities. For instance, Si_24_-based electrodes could be interesting for all-solid state microbatteries for aerospace, medical implants, stand-alone microelectronic devices, nanometer-sized autonomous sensors, and so on. Furthermore, the physical qualities predicted for Na_*x*_Si_24_ make interesting to explore the insertion of other ions into open framework silicon compounds for their potential use, for instance, in K- and Mg-ion batteries.

## Electronic supplementary material

Supplementary Information

## References

[1] Tarascon J-M (2010). Is lithium the new gold?. Nat. Chem.

[2] Slater MD, Kim D, Lee E, Johnson CS (2013). Sodium-Ion Batteries. Adv. Funct. Mater..

[3] Kundu D, Talaie E, Duffort V, Nazar LF (2015). The Emerging Chemistry of Sodium Ion Batteries for Electrochemical Energy Storage. Angew. Chem., Int. Ed..

[4] Palomares V (2012). Na-ion batteries, recent advances and present challenges to become low cost energy storage systems. Energy Environ. Sci..

[5] Kim S-W, Seo D-H, Ma X, Ceder G, Kang K (2012). Electrode Materials for Rechargeable Sodium-Ion Batteries: Potential Alternatives to Current Lithium-Ion Batteries. Adv. Energy Mater..

[6] Hueso KB, Armand M, Rojo T (2013). High temperature sodium batteries: status, challenges and future trends. Energy Environ. Sci..

[7] Yabuuchi N, Kubota K, Dahbi M, Komaba S (2014). Research Development on Sodium-Ion Batteries. Chem. Rev..

[8] Pan H, Hu Y-S, Chen L (2013). Room-temperature stationary sodium-ion batteries for large-scale electric energy storage. Energy Environ. Sci..

[9] Han MH, Gonzalo E, Singh G, Rojo T (2015). A comprehensive review of sodium layered oxides: powerful cathodes for Na-ion batteries. Energy Environ. Sci..

[10] Landa-Medrano I (2016). Sodiumâ“Oxygen Battery: Steps Toward Reality. J. Phys. Chem. Lett..

[11] Luo W (2016). Na-Ion Battery Anodes: Materials and Electrochemistry. Acc. Chem. Res..

[12] Kim H (2016). Recent Progress in Electrode Materials for Sodium-Ion Batteries. Adv. Energy Mater..

[13] Nitta N, Wu F, Lee JT, Yushin G (2015). Li-ion battery materials: present and future. Mater. Today.

[14] Yazami R, Touzain P (1983). A reversible graphite-lithium negative electrode for electrochemical generators. J. Power Sources.

[15] Winter M, Besenhard JO, Spahr ME, NovÃ¡k P (1998). Insertion Electrode Materials for Rechargeable Lithium Batteries. Adv. Mater..

[16] Ge P (1988). Electrochemical intercalation of sodium in graphite. Solid State Ion.

[17] Jache B, Adelhelm P (2014). Use of Graphite as a Highly Reversible Electrode with Superior Cycle Life for Sodium-Ion Batteries by Making Use of Co-Intercalation Phenomena. Angew. Chem. Int. Ed..

[18] Hatchard TD, Dahn JR (2004). *In Situ* XRD and Electrochemical Study of the Reaction of Lithium with Amorphous Silicon. J. Electrochem. Soc..

[19] Liu XH (2012). *In situ* atomic-scale imaging of electrochemical lithiation in silicon. Nat. Nanotechnol..

[20] Key B, Morcrette M, Tarascon J-M, Grey CP (2011). Pair Distribution Function Analysis and Solid State NMR Studies of Silicon Electrodes for Lithium Ion Batteries: Understanding the (De)lithiation Mechanisms. J. Am. Chem. Soc..

[21] Komaba S (2012). Redox reaction of Sn-polyacrylate electrodes in aprotic Na cell. Electrochem. Commun..

[22] Ellis LD, Wilkes BN, Hatchard TD, Obrovac MN (2014). *In Situ* XRD Study of Silicon, Lead and Bismuth Negative Electrodes in Nonaqueous Sodium Cells. J. Electrochem. Soc..

[23] Lim C-H (2016). Experimental Study on Sodiation of Amorphous Silicon for Use as Sodium-Ion Battery Anode. Electrochim. Acta.

[24] Zhao Q (2016). A Si/C nanocomposite anode by ball milling for highly reversible sodium storage. Electrochem. Commun..

[25] Xu Y (2016). Reversible Na-Ion Uptake in Si Nanoparticles. Adv. Energy Mater.

[26] Zhang Y (2017). In operando mechanism analysis on nanocrystalline silicon anode material for reversible and ultrafast sodium storage. Adv. Mater..

[27] Zhang L (2004). Electrochemical performance of lithium ion battery, nano-silicon-based, disordered carbon composite anodes with different microstructures. J. Power Sources.

[28] Szczech JR, Jin S (2011). Nanostructured silicon for high capacity lithium battery anodes. Energy Environ. Sci..

[29] McDowell MT, Lee SW, Nix WD, Cui Y (2013). 25th Anniversary Article: Understanding the Lithiation of Silicon and Other Alloying Anodes for Lithium-Ion Batteries. Adv. Mater..

[30] Chou C-Y, Lee M, Hwang GS (2015). A Comparative First-Principles Study on Sodiation of Silicon, Germanium, and Tin for Sodium-Ion Batteries. J. Phys. Chem. C.

[31] Obrovac MN, Christensen L (2004). Structural Changes in Silicon Anodes during Lithium Insertion/Extraction. Electrochem. Solid State Lett..

[32] Morito H, Yamada T, Ikeda T, Yamane H (2009). Naâ“Si binary phase diagram and solution growth of silicon crystals. J. Alloys Compd..

[33] Conesa JC (2002). Computer Modeling of *allo*-Si and *allo*-Ge Polymorphs. J. Phys. Chem. B.

[34] Haberl B, Strobel TA, Bradby JE (2016). Pathways to exotic metastable silicon allotropes. Appl. Phys. Rev.

[35] Taylor PC (2016). Exotic forms of silicon. Phys. Today.

[36] Beekman M, Wei K, Nolas GS (2016). Clathrates and beyond: Low-density allotropy in crystalline silicon. Appl. Phys. Rev..

[37] Warrier P, Koh CA (2016). Silicon clathrates for lithium ion batteries: A perspective. Appl. Phys. Rev..

[38] Kim DY, Stefanoski S, Kurakevych OO, Strobel TA (2014). Synthesis of an open-framework allotrope of silicon. Nat. Mater..

[39] Kasper JS, Hagenmuller P, Pouchard M, Cros C (1965). Clathrate Structure of Silicon Na8si46 and NaxSi136 (x < 11). Science.

[40] Slingsby JG (2016). Dynamic free energy surfaces for sodium diffusion in type II silicon clathrates. Phys. Chem. Chem. Phys..

[41] Moriguchi K, Yonemura M, Shintani A, Yamanaka S (2000). Electronic structures of Na8Si46 and Ba8Si46. Phys. Rev. B.

[42] Kurakevych OO, Strobel TA, Kim DY, Muramatsu T, Struzhkin VV (2013). Na-Si Clathrates Are High-Pressure Phases: A Melt-Based Route to Control Stoichiometry and Properties. Cryst. Growth Des..

[43] Marzouk A, Balbuena PB, El-Mellouhi F (2016). Open Framework Allotropes of Silicon: Potential Anode Materials for Na and Li-ion Batteries. Electrochim. Acta.

[44] Kresse G, FurthmÃller J (1996). Efficient iterative schemes for *ab initio* total-energy calculations using a plane-wave basis set. Phys. Rev. B.

[45] Kresse G, FurthmÃller J (1996). Efficiency of ab-initio total energy calculations for metals and semiconductors using a plane-wave basis set. Comput. Mater. Sci..

[46] Kresse G, Joubert D (1999). From ultrasoft pseudopotentials to the projector augmented-wave method. Phys. Rev. B.

[47] Perdew JP, Burke K, Ernzerhof M (1996). Generalized Gradient Approximation Made Simple. Phys. Rev. Lett..

[48] Van der Ven, A., Thomas, J. C., Xu, Q., Swoboda, B. & Morgan, D. Nondilute diffusion from first principles: Li diffusion in LixTiS2. *Phys*. *Rev*. *B* **78** (2008).

[49] Henkelman G, Uberuaga BP, JÃnsson H (2000). A climbing image nudged elastic band method for finding saddle points and minimum energy paths. J. Chem. Phys..

[50] Carrasco J (2014). Role of van der Waals Forces in Thermodynamics and Kinetics of Layered Transition Metal Oxide Electrodes: Alkali and Alkaline-Earth Ion Insertion into V_2_O_5_. J. Phys. Chem. C.

[51] Rao D (2017). Ultrahigh energy storage and ultrafast ion diffusion in borophene-based anodes for rechargeable metal ion batteries. J. Mater. Chem. A.

[52] Grimme S, Ehrlich S, Goerigk L (2011). Effect of the damping function in dispersion corrected density functional theory. J. Comput. Chem..

[53] Lozano A, Escribano B, Akhmatskaya E, Carrasco J (2017). Assessment of van der Waals inclusive density functional theory methods for layered electroactive materials. Phys. Chem. Chem. Phys..

[54] Aydinol MK, Kohan AF, Ceder G, Cho K, Joannopoulos J (2011). *Ab initio* study of lithium intercalation in metal oxides and metal dichalcogenides. Phys. Rev. B.

[55] Jung SC, Jung DS, Choi JW, Han Y-K (2014). Atom-Level Understanding of the Sodiation Process in Silicon Anode Material. J. Phys. Chem. Lett..

[56] Baggetto L (2013). Intrinsic thermodynamic and kinetic properties of Sb electrodes for Li-ion and Na-ion batteries: experiment and theory. J. Mater. Chem. A.

[57] Qian J (2012). High capacity Na-storage and superior cyclability of nanocomposite Sb/C anode for Na-ion batteries. Chem. Comm..

[58] Tao D (2016). First-principles study of Na_2+*x*_Ti_7_O_15_ as anode materials for sodium-ion batteries. J. Alloys Compd..

[59] Rousse G (2013). Rationalization of Intercalation Potential and Redox Mechanism for A_2_Ti_3_O_7_ (A = Li, Na). Chem. Mater..

[60] Fullenwarth J, Darwiche A, Soares A, Donnadieu B, Monconduit L (2014). NiP_3_: a promising negative electrode for Li- and Na-ion batteries. J. Mater. Chem. A.

[61] Zhao L (2012). Disodium Terephthalate (Na2C8H4O4) as High Performance Anode Material for Low-Cost Room-Temperature Sodium-Ion Battery. Adv. Energy Mater.

[62] Abouimrane A (2012). Sodium insertion in carboxylate based materials and their application in 3.6 V full sodium cells. Energy Environ. Sci..

[63] Castillo-MartÃnez E, Carretero-GonzÃ¡lez J, Armand M (2014). Polymeric Schiff Bases as Low-Voltage Redox Centers for Sodium-Ion Batteries. Angew. Chem., Int. Ed..

[64] LÃpez-Herraiz M (2015). Oligomeric-Schiff bases as negative electrodes for sodium ion batteries: unveiling the nature of their active redox centers. Energy Environ. Sci..

[65] Kulish VV (2014). Controlling Na diffusion by rational design of Si-based layered architectures. Phys. Chem. Chem. Phys..

[66] Deng Z, Mo Y, Ong SP (2016). Computational studies of solid-state alkali conduction in rechargeable alkali-ion batteries. NPG Asia Mater..

[67] Yabuuchi N (2014). Phosphorus Electrodes in Sodium Cells: Small Volume Expansion by Sodiation and the Surface-Stabilization Mechanism in Aprotic Solvent. ChemElectroChem.

[68] Duveau D, Israel SS, Fullenwarth J, Cunin F, Monconduit L (2016). Pioneer study of SiP_2_ as negative electrode for Li- and Na-ion batteries. J. Mater. Chem. A.

