# Supplementary material for: First-Principles Study of Sodium Intercalation in Crystalline Nax Si24 (0 ≤ x ≤ 4) as Anode Material for Na-ion Batteries
Source: Sci Rep. 2017 Jul 13;7:5350. doi: 10.1038/s41598-017-05629-x (PMC5509687; doi:10.1038/s41598-017-05629-x)
Supplement: Supplementary file 1 — Supplementary Information [file 41598_2017_5629_MOESM1_ESM.pdf]

Supplementary Information

First-Principles Study of Sodium Intercalation in Crystalline Na_xSi_{24} ($0 \le x \le 4$) as Anode Material for Na-ion Batteries

Unai Arrieta[1], Nebil A. Katcho[1], Oier Arcelus[1], and Javier Carrasco[1,*]

[1] CIC Energigune, Parque Tecnológico de Álava, C/ Albert Einstein 48, 01510 Miñano (Vitoria, Álava), Spain

* jcarrasco@cicenergigune.com

Table S1. Computed formation energies, optimized cell parameters, and cell volume for ground state and second most stable Na_xSi_{24} structures.

x in Na_xSi_{24}	$\Delta_f E$ (meV/$Na_{x/4}Si_6$)	*a* (Å)	*b* (Å)	*c* (Å)	α (º)	β (º)	γ (º)	Volume (Å^3/$Na_{x/4}Si_6$)
0.000	0.0	3.8521	10.7501	12.7422	90.00	90.00	90.00	131.92
0.250	-5.5	7.7441	21.4690	12.7161	90.00	90.00	90.00	132.14
0.333	-6.3	10.7309	11.6370	13.2837	106.97	89.99	90.00	132.21
0.333	2.2	3.8761	10.7364	38.1285	90.00	90.00	89.99	132.23
0.500	-12.1	7.7863	21.4342	12.6881	90.00	90.00	90.00	132.35
0.500	-5.4	7.7748	10.7301	13.2759	90.00	72.97	90.00	132.37
0.667	-2.5	11.4142	11.6856	13.2709	72.94	84.24	70.05	132.56
0.667	-2.3	3.9034	16.5931	24.8984	99.63	90.00	90.00	132.50
1.000	-1.8	3.9219	10.7094	12.6480	90.00	90.00	89.99	132.81
1.000	-0.2	7.8499	10.7127	13.2368	90.00	72.75	90.00	132.89
1.333	1.2	10.6848	11.8696	13.2009	107.42	89.99	89.99	133.12
1.333	2.9	11.3943	11.8582	12.6018	90.00	90.00	110.28	133.09
1.500	2.7	7.9387	11.3908	12.5808	90.00	90.00	110.39	133.29
1.500	3.5	7.9357	11.3919	13.1932	83.98	72.52	69.62	133.30
1.667	5.1	10.6681	11.9472	12.5591	90.00	90.00	89.99	133.39
1.667	5.5	11.3900	11.9449	12.5584	90.00	90.00	110.44	133.42
2.000	2.2	8.0105	11.3903	12.5266	90.00	90.00	110.63	133.70
2.000	5.3	11.3908	12.0031	12.5298	90.00	90.00	110.57	133.66
2.333	5.9	11.4009	12.0285	12.5173	90.00	90.00	110.59	133.91
2.333	6.5	11.3961	12.0469	12.5085	90.00	90.00	110.63	133.92
2.500	9.9	8.0504	11.3957	12.4940	90.00	90.00	110.67	134.05
2.500	9.9	8.0448	11.3995	13.1300	83.78	72.15	69.33	134.05
2.667	6.9	11.4060	12.0743	12.4946	90.00	90.00	110.66	134.17
2.667	7.3	10.6700	12.0704	13.1298	107.86	90.00	90.00	134.12
3.000	9.1	10.6813	12.0982	12.4811	90.00	90.00	89.98	134.41
3.000	9.6	10.6771	12.1045	12.4769	90.00	90.00	89.99	134.38
3.333	4.3	11.4202	12.1307	12.4650	90.00	90.00	110.72	134.59
3.333	8.8	11.4229	12.1205	12.4696	90.00	90.00	110.70	134.58
3.500	7.9	16.3590	10.6299	12.3911	90.00	90.00	90.00	134.67
3.500	10.0	8.0725	10.6987	13.1135	90.00	72.07	90.00	134.70
3.667	6.3	10.7004	12.1279	13.1125	107.96	89.99	90.00	134.89
4.000	0.0	4.1893	10.5376	12.2094	90.00	90.00	90.00	134.75

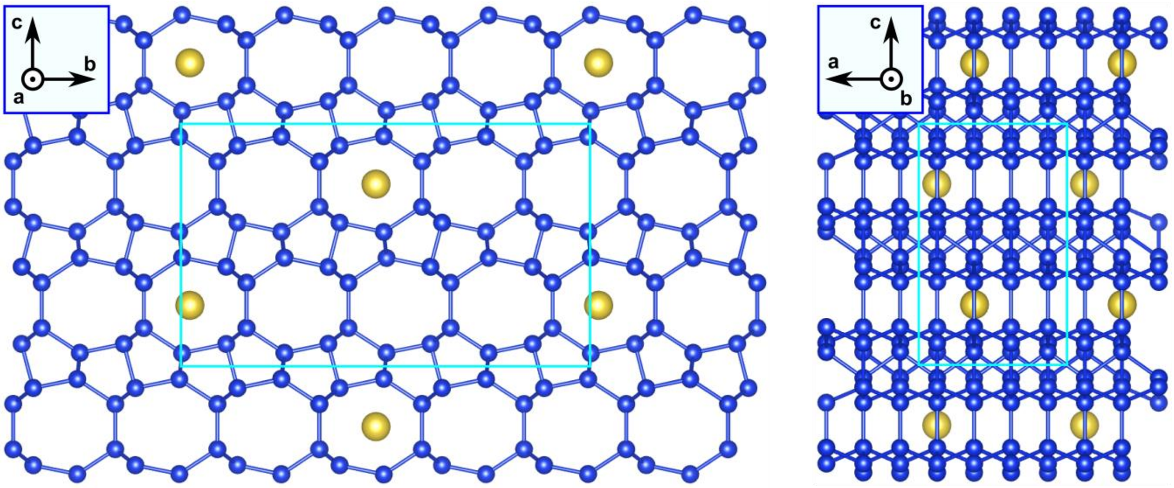

Figure S1. Optimized structure of the most stable $Na_{0.5}Si_{24}$ structure projected in b-c and a-c planes. The light blue rectangle shows the unit cell, which contains 16 $Na_{0.125}Si_6$ formula units. Blue and yellow balls stand for silicon and sodium atoms, respectively.
